# Inflammation and its associations with aortic stiffness, coronary artery disease and peripheral artery disease in different ethnic groups: The HELIUS Study

**DOI:** 10.1016/j.eclinm.2021.101012

**Published:** 2021-07-07

**Authors:** Charles F. Hayfron-Benjamin, Charlotte Mosterd, Anke H. Maitland - van der Zee, Daniel H. van Raalte, Albert G.B. Amoah, Charles Agyemang, Bert-Jan van den Born

**Affiliations:** a Department of Internal and Vascular Medicine, Amsterdam UMC, University of Amsterdam, Cardiovascular Sciences, Amsterdam, the Netherlands; b Department of Public Health, Amsterdam UMC, University of Amsterdam, Amsterdam Public Health Research Institute, Amsterdam, the Netherlands; c Department of Respiratory Medicine, Amsterdam UMC, University of Amsterdam, Amsterdam, the Netherlands; d Department of Medicine and Therapeutics, University of Ghana Medical School, Ghana; e Department of Physiology, University of Ghana Medical School, Ghana

**Keywords:** Inflammation, Coronary Artery Disease, Peripheral Artery Disease, Aortic Stiffness, Cardiovascular Disease, Race and Ethnicity

## Abstract

**Background:**

evidence shows important ethnic differences in vascular dysfunction rates; however, the mechanisms driving these differences remain unclear. One potential factor is the ethnic differences in the role of inflammation in vascular injury. We tested the hypothesis that low-grade inflammation is unequally associated with vascular dysfunction in different ethnic groups.

**Methods:**

we included 5698 participants (similar-sized Dutch, African Surinamese, South-Asian Surinamese, Ghanaians, Turkish, and Moroccans) of the HELIUS study (the Netherlands) conducted between 2011 and 2015. Logistic regression was used to examine the associations of Z-score inflammatory biomarker concentration (high sensitivity C-reactive protein [hs-CRP], fibrinogen, and d-dimer) with vascular dysfunction (aortic stiffness, coronary artery disease [CAD], and peripheral artery disease [PAD]), with adjustments for age, sex, smoking (pack-years), BMI, hypertension, HbA1c, total cholesterol, and statin use

**Findings:**

in the fully adjusted models, higher Z-score hs-CRP was positively associated with CAD in Dutch [OR 1·63, (95% CI 1·21–2·18)] and PAD in South Asians [1·25(1·03–1·53)], respectively. Higher Z-score fibrinogen was positively associated with CAD in African Surinamese [1·28(1·03–1·59)] while higher Z-score d-dimer was positively associated with PAD in Moroccans [1·39(1·01–1·93)]. Higher Z-score hs-CRP [0·71(0·54–0·94)] and fibrinogen [0·75(0·58–0·97)] concentrations were negatively associated with PAD in African Surinamese.

**Interpretation:**

our study shows that inflammatory biomarkers are unequally associated with vascular dysfunction in different ethnic groups. These observations provide opportunities for future studies aimed at assessing the predictive roles of inflammation on vascular disease in different ethnic groups.

**Research in context**

**Evidence before this study**

We searched PubMed for articles published in English using the following search terms, ‘ethnicity’, ‘race’, ‘inflammation’ ‘low-grade inflammation’, ‘C reactive protein’, ‘fibrinogen’, ‘D-dimer’, ‘cardiovascular disease’, ‘vascular dysfunction’, ‘aortic stiffness’, ‘coronary artery disease, ‘coronary heart disease’, ‘peripheral vascular disease’ and ‘peripheral artery disease’ before June 2020. Through the searches, we found ethnic differences in the rates of coronary artery disease, peripheral artery disease, and aortic stiffness, which were not sufficiently explained by the conventional cardiometabolic risk factors. We also found reports of ethnic differences in the levels of inflammatory-related biomarkers. Some reports had linked inflammation or low-grade inflammation to cardiovascular disease risk. However, these reports were in multiethnic populations and were not stratified by ethnicity

**Added value of this study**

Using a multiethnic cohort living in Amsterdam, we determined the associations of three inflammatory biomarkers (high sensitivity C-reactive protein, fibrinogen, and D-dimer) with aortic stiffness, coronary artery disease, and peripheral artery disease in different ethnic groups. After adjusting for a wide range of conventional cardiometabolic risk factors, we found important ethnic differences in the associations of low-grade inflammation and different measures of vascular dysfunction.

**Implications of all the available evidence**

Our findings provide opportunities for future studies aimed at assessing the predictive roles of inflammatory biomarkers on vascular disease in different ethnic groups. This may aid vascular disease risk prevention and/or treatment efforts in different ethnic groups.

Alt-text: Unlabelled box

## 1 Introduction

Worldwide, atherothrombotic vascular diseases such as coronary artery disease (CAD) and peripheral artery disease (PAD) are highly prevalent and frequently complicated by acute coronary syndromes and critical limb ischemia, respectively [1,2]. Likewise, aortic stiffness, a surrogate endpoint to macrovascular dysfunction, is common and is associated with systolic hypertension, heart failure, and atrial fibrillation [3,4]. These large vessel-related diseases contribute to repeated hospitalizations and drive mortality including sudden cardiac death [1,3,4].

Existing data suggest important ethnic differences in the burden of vascular diseases; however, the mechanisms driving these differences are not fully understood [5], [6], [7]. The conventional vascular risk factors based on the cardiometabolic hypothesis, including cigarette smoking, hypertension, dyslipidemia, and diabetes, are in themselves unable to sufficiently explain these differences [8]. One potential modifiable risk factor is the differential roles of systemic or vascular inflammation on vascular dysfunction [9]. Previous studies have highlighted important ethnic differences in the level of circulating inflammatory biomarkers and clotting activation factors inherently linked with inflammation, which may be partially related to demographic, lifestyle, or genetic factors [10], [11], [12]. A previous study has reported that the risk of vascular dysfunction in similar individuals with similar levels of inflammation varied by ethnicity [13]. Given the above and the evidence of inflammation as a key pathogenic mechanism in atherogenesis [9], it is biologically plausible that the role of inflammation in vascular dysfunction may vary by ethnicity. However, data on the role of ethnic differences in the associations between inflammation and vascular dysfunction are lacking. We tested the hypothesis that low-grade inflammation assessed by elevated concentration of three inflammatory biomarkers (high sensitivity C-reactive protein [hs-CRP], fibrinogen, and D-dimer) is unequally associated with prevalent aortic stiffness, CAD, and PAD in six different ethnic groups resident in Amsterdam, the Netherlands.

## 2 Methods

### 2.1 Data statement

The Healthy Life in an Urban Setting (HELIUS) data are owned by the Academic Medical Center (AMC) in Amsterdam, The Netherlands. Any researcher can request the data by submitting a proposal to the HELIUS Executive Board as outlined at http://www.heliusstudy.nl/en/researchers/collaboration. Charles F. Hayfron-Benjamin, Lot Mosterd, Charles Agyemang, and Bert-Jan van den Born had access to the data during the study.

### 2.2 Study design

The current study is a cross-sectional analysis of baseline data of the HELIUS cohort. The baseline data from the HELIUS study was collected between 2011 and 2015. The rationale, study design, cohort description, and methodology of the HELIUS study have been described in detail elsewhere [14,15]. In brief, HELIUS was a multi-ethnic prospective cohort study among six large ethnic groups in Amsterdam [Dutch (Western Europe), African Surinamese (South America with African roots), Ghanaian (West Africa), Moroccan (North Africa), South-Asian Surinamese (Indian subcontinent), and Turkish ethnic origin]. The ethnic minority groups included in the HELIUS study are the largest ethnic minority groups in Amsterdam [16]. The study participants were randomly sampled from the municipality registry, stratified by ethnicity. Ethnicity was defined by the individual's country of birth combined with the parental countries of birth [17]. Non-Dutch ethnic origin was assigned to participants born abroad with at least one parent born abroad or born in the Netherlands with both parents born abroad.

Data were collected among 24,789 participants; questionnaires, physical examinations, and biological samples were obtained. The concentrations of inflammatory biomarkers were measured in random subsamples of 1000 participants from each ethnic group (total *n* = 6000), who had complete data on cardiovascular measurements and had stored blood samples available for measurements of the concentrations of inflammatory biomarkers. Inflammatory biomarkers assessed were hs-CRP, fibrinogen, and D-dimer. We selected these biomarkers because they are well-established markers of inflammation, with well-characterized clinical endpoints [18,19]. Additionally, these biomarkers are known to predict future cardiovascular events [19,20]. Because our interest was in low-grade inflammation, we excluded participants with CRP levels above 10 mg/L (*n* = 250), as this can indicate acute inflammation [9]. Further, we excluded individuals with incomplete data on measures of vascular function (*n* = 52). Therefore, we included 5698 participants (963 Dutch, 949 South-Asian Surinamese, 941 African Surinamese, 962 Ghanaian, 946 Turkish, and 937 Moroccan participants) in the current analyses.

The study was approved by the Ethics Committee of the Amsterdam Medical Center (MREC 10/100# 17.10.1729) before data collection and all participants provided written informed consent. The study protocol conforms to the ethical guidelines of the 1975 Declaration of Helsinki as reflected in a priori approval by the institution's human research committee. The data, analytic methods, and study materials can be made available to other researchers for purposes of reproducing the results or replicating the procedure, after completion of a research proposal to the authors and the HELIUS scientific coordinator, including a data use agreement, and only after approval by the HELIUS executive board.

### 2.3 Assessments

A structured questionnaire was used to record the demographic, socioeconomic, and health-related behaviors of the study participants. Smoking status was classified into nonsmokers and current smokers and the number of pack-years was calculated by multiplying the number of packs (containing 20 cigarettes) smoked a day by the number of years. Weight was measured in light clothing and without shoes with SECA 877 scales. Height was measured without shoes with SECA 217 stadiometer. Body mass index (BMI) was determined from weight and height. Blood pressure (BP) was measured thrice using the Microlife Watch BP home device, with appropriately sized cuffs after at least 5 min rest while seated. The mean of the last two BP measurements was used for the analyses. Hypertension was defined as systolic blood pressure ≥140 mm Hg and/or a diastolic blood pressure ≥ 90 mm Hg and/or current use of antihypertensive agents.

Ankle-brachial pressure index (ABI)measurements were performed in the supine position using a validated oscillometric device (Microlife WatchBP Office ABI, Switzerland) with appropriate-sized cuffs, after at least 10 min of supine rest [21]. Systolic BP was measured twice in the right and left brachial arteries and twice in the right and left posterior tibial arteries. ABI was calculated by taking the highest arm systolic BP as the denominator, and the lowest ankle BP as the numerator [22]. The lowest of the left and right ABI measurements was used for analyses. ABI obtained by the oscillometric method using the Microlife WatchBP Office ABI has been shown to correlate well with ABI acquired by Doppler ultrasound with a 95% agreement between the two methods in diagnosing PAD [23]. Aortic stiffness measurements were performed in duplicate after 10 min of supine rest using the Arteriograph system (Tensiomed Kft, Hungary) and the mean of these two measurements was used for analyses. The details of the aortic stiffness measurements are described elsewhere [5]. Among other indices, the aortic pulse wave velocity (AoPWV) was measured. PWV measured by the Arteriograph system generates similar PWV values as obtained by Magnetic Resonance Imaging [24]. Standard 12-lead supine digital resting electrocardiography (ECG) was recorded (GE MAC5500, 500 samples/sec) and processed with the Modular ECG Analysis System (MEANS) program. MEANS determines common P-wave, QRS, and T-wave onsets and offsets for all 12 leads together on one representative averaged beat. All on- and offsets were manually checked and adjusted when necessary.

#### 2.3.1 Biochemical analyses

Fasting blood samples were drawn and plasma samples were used to determine the concentration of glucose by spectrophotometry, using hexokinase as the primary enzyme (Roche Diagnostics, Japan). Diabetes mellitus was defined by fasting plasma glucose concentration of ≥ 7.0 mmol/l and/or HbA1c ≥48 mmol/mol and/or the use of glucose-lowering agents. Total cholesterol, triglycerides, and HDL cholesterol were determined by colorimetric spectrophotometry. LDL cholesterol was calculated according to the Friedewald formula [25]. Glycated hemoglobin (HbA1c) was measured by high-performance liquid chromatography technology (TOSOH, Tokyo, Japan). The hs-CRP concentration was measured in heparin plasma by a particle enhanced immunoturbidimetric assay. Human CRP agglutinates with latex particles were coated with monoclonal anti-CRP antibodies. The aggregates were determined turbidimetrically with a Cobas 702c analyzer (Roche Diagnostics, Mannheim, Germany). In 622 participants, the CRP-value was below the detection limit (<0.3  mg/L) and replaced by a value of 0.15  mg/L. D-dimer concentrations were quantified by commercially available ELISA kits. Fibrinogen levels were determined with the immunoprecipitation test.

#### 2.3.2 Definition of vascular dysfunction

PAD was defined as ABI ≤0.90 [22]. In defining normal ABI, an ABI > 1.4 (*n* = 18) was excluded as it could be suggestive of non-compressible vessels [22]. CAD was based on the Rose Angina Questionnaire [26] and/or the presence of pathological Q waves in at least 2 contiguous leads on ECG. ST-segment abnormalities were not included in the definition of CAD due to the highly variable ethnicity‐dependent prevalence of ECGs with ST‐segment elevations exceeding STEMI thresholds in apparently healthy individuals [27]. Large artery stiffness was based on aortic stiffness, defined as AoPWV greater than 12 m/s [28].

### 2.4 Statistical analysis

Data were analyzed using the IBM SPSS version 23 for Windows. We first verified whether the association between inflammatory biomarkers and vascular dysfunction differed by ethnicity. We stratified the analyses by ethnicity, as significant interaction effects (ethnicity*inflammatory markers) were found for at least one inflammatory biomarker per vascular complication. We also verified whether the association between inflammatory biomarkers and vascular dysfunction differed by sex. Significant interaction was found for the association between aortic stiffness and hs-CRP and fibrinogen in Dutch, fibrinogen, and D-dimer in South Asian Surinamese, and fibrinogen and D-dimer in Turkish participants. In a supplementary analysis, we stratified the analysis by sex, where relevant. Data with normal distributions were presented as mean (± standard deviation), whereas those not normally distributed were presented as median (interquartile range). For normally distributed data, Analysis Of Variance (ANOVA) was used to compare means among ethnic groups. For data not normally distributed, the Kruskal-Wallis ANOVA test was used to compare medians among ethnic groups. Categorical data were presented as frequencies (percentages). Logistic regression analyses were used to examine the associations between Inflammatory biomarker concentration and vascular dysfunction, with adjustments for covariates. In a supplementary analysis, we assessed these associations based on clinically recommended cut-off points: the concentrations of hs-CRP, fibrinogen, and D-dimer were considered elevated if above 3 mg/L, 3.5 g/L, and 0.55 mg/L, respectively [29], [30], [31]. Odds ratios (ORs) and their corresponding 95% CI were estimated. The minimal sufficient adjustment sets for estimating the direct effect of inflammation on vascular dysfunction were determined by a directed acyclic graph (DAG) (DAG available at dagitty.net/mFv38Kd). DAG was chosen because the traditional methods of adjusting for potential confounders can introduce conditional associations and bias rather than minimize it [32]. Based on the DAG, the minimal sufficient adjustment sets for estimating the total effect of inflammation on vascular dysfunction were age, sex, smoking, obesity, and the presence of hypertension, diabetes, and dyslipidemia. Two models were used to examine the data. Model 1 was unadjusted; Model 2 was adjusted for age, sex, smoking (pack-years), BMI, hypertension, HbA1c, total cholesterol concentration, and statin use. A statistical test of significance was set at a *p*-value < 0.05.

### 2.5 Role of the funding source

The funding sources had no involvement in the study design, data collection, analysis, or interpretation

## 3 Results

### 3.1 General characteristics

The baseline characteristics of the study population are shown in Table 1. Dutch participants had the highest socioeconomic status and the lowest BMI but had the highest median pack-years of smoking. The South-Asian Surinamese group had the highest prevalence of diabetes and the highest mean HnA1c and LDL-cholesterol concentrations, while the African Surinamese group had the highest proportion of smokers but were the most physically active. Ghanaians had the highest mean systolic and diastolic blood pressures but had the lowest smoking indices. The Dutch group has the lowest rates of CAD, PAD, and large artery stiffness, while South Asian Surinamese participants had the highest rates of CAD, PAD, and large artery stiffness.

**Table 1:** Characteristics of the study population, stratified by ethnic background.

	Dutch	South-Asian Surinamese	African Surinamese	Ghanaian	Turkish	Moroccan
N	963	949	941	962	946	937
Male sex, (%)	462 (48.0)	477 (50.2)	378 (40.1)	411 (42.7)	423 (44.6)	328 (35.0)
Age, years	45.14 (±13.90)	46.54 (±13.51)	47.66 (±12.50)	45.61 (±11.18)	40.57 (±11.96)	40.70 (±13.06)
First-generation migrant (%)	Not applicable	749 (78.8)	792 (84.1)	921 (95.7)	676 (71.3)	649 (69.2)
Higher education (%)	594 (61.7)	228 (24.0)	244 (25.9)	68 (7.1)	144 (15.2)	155 (16.5)
Smoking status (%)						
Never smokers	342 (35.5%)	538 (56.7%)	458 (48.7%)	828 (86.1%)	438 (46.3%)	706 (75.3%)
Previous smokers	379 (39.4%)	140 (14.8%)	183 (19.4%)	83 (8.6%)	197 (20.8%)	109 (11.6%)
Current smokers	242 (25.1%)	271 (28.6%)	300 (31.9%)	51 (5.3%)	311 (32.9%)	122 (13.0%)
Smoking (pack-years)*	1.20 (12.00)	0.00 (5.02)	0.00 (6.00)	0.00 (0.00)	0.33 (9.47)	0.00 (0.00)
BMI, kg/m^2^	24.44 (±3.75)	25.80 (±4.20)	27.04 (±4.97)	28.27 (±4.47)	27.88 (±5.09)	27.12 (±4.89)
Systolic BP, mmHg	124.37 (±16.16)	130.19 (±18.35)	131.41 (±17.39)	136.53 (±18.45)	123.89 (±16.01)	121.55 (±15.52)
Diastolic BP, mmHg	76.50 (±9.95)	80.31 (±10.64)	81.32 (±10.21)	84.87 (±11.34)	77.52 (±10.14)	73.93 (±9.42)
Cardiovascular traits						
Hypertension (%)	232 (24.1)	393 (41.4)	405 (43.0)	526 (54.7)	243 (25.6)	161 (17.2)
Diabetes (%)	34 (3.5%)	204 (21.5%)	119 (12.6%)	125 (13.0%)	88 (9.3%)	117 (12.5%)
Medication use						
Antithrombotic drugs (%)	47 (4.9)	88 (9.3)	42 (4.5)	21 (2.2)	32 (3.4)	13 (1.4)
Systemic steroids (%)	2 (0.2)	3 (0.3)	5 (0.5)	4 (0.4)	5 (0.5)	2 (0.2)
Statins (%)	82 (8.5)	206 (21.7)	70 (7.4)	83 (8.6)	81 (8.5)	76 (8.1)
Biochemical						
HbA1c, mmol/mol	36.31 (±4.61)	42.80 (±10.20)	40.18 (±9.01)	39.19 (±8.68)	38.71 (±7.87)	39.45 (±8.63)
Total cholesterol, mmol/l	5.10 (±1.04)	4.92 (±1.05)	4.88 (±0.97)	4.98 (±1.02)	4.87 (±0.99)	4.59 (±0.94)
LDL-cholesterol, mmol/l	3.07 (±0.94)	3.09 (±0.94)	2.97 (±0.90)	3.02 (±0.92)	3.02 (±0.87)	2.81 (±0.84)
Elevated inflammation biomarker concentration						
High hs-CRP (>3 mg/L) (%)	121 (12.6)	232 (24.4)	209 (22.3)	165 (17.2)	243 (25.7)	246 (26.3)
High Fibrinogen (> 3.5 g/L) (%)	50 (5.2)	164 (17.3)	176 (18.7)	105 (11.0)	94 (9.9)	128 (13.7)
High d-dimer (0.55 mg/L) (%)	52 (5.4)	79 (8.3)	134 (14.2)	95 (9.9)	63 (6.7)	62 (6.6)
Macrovascular function						
AoPWV (m/s)	7.93 (±2.16)	8.69 (±2.55)	8.51 (±2.27)	8.65 (±2.19)	8.08 (±2.19)	7.84 (±2.25)
Aortic stiffness (%)	55 (5.7%)	114 (12.0%)	92 (9.9%)	80 (8.3%)	71 (7.5%)	68 (7.3%)
ABI	1.15 (±0.13)	1.10 (±0.14)	1.12 (±0.14)	1.11 (±0.14)	1.11 (±0.15)	1.13 (±0.13)
Peripheral artery dsease (%)	45 (4.7%)	108 (11.4%)	83 (8.8%)	82 (8.5%)	96 (10.1%)	63 (6.7%)
Coronary artery disease (%)	48 (5.0%)	171 (18.0%)	120 (12.8%)	107 (11.1%)	189 (20.0%)	171 (18.2%)

Data are mean (±standard deviation), median (IQR), or n (%).

Abbreviations: ABI = ankle-brachial pressure index, AoPWV = aortic pulse wave velocity, BMI = body mass index; hs-CRP = high sensitivity C-reactive protein, LDL = low density lipoprotein.

* Data presented as median (interquartile range).

### 3.2 Concentrations of inflammatory biomarkers in different ethnic groups

**Fig. 1:**
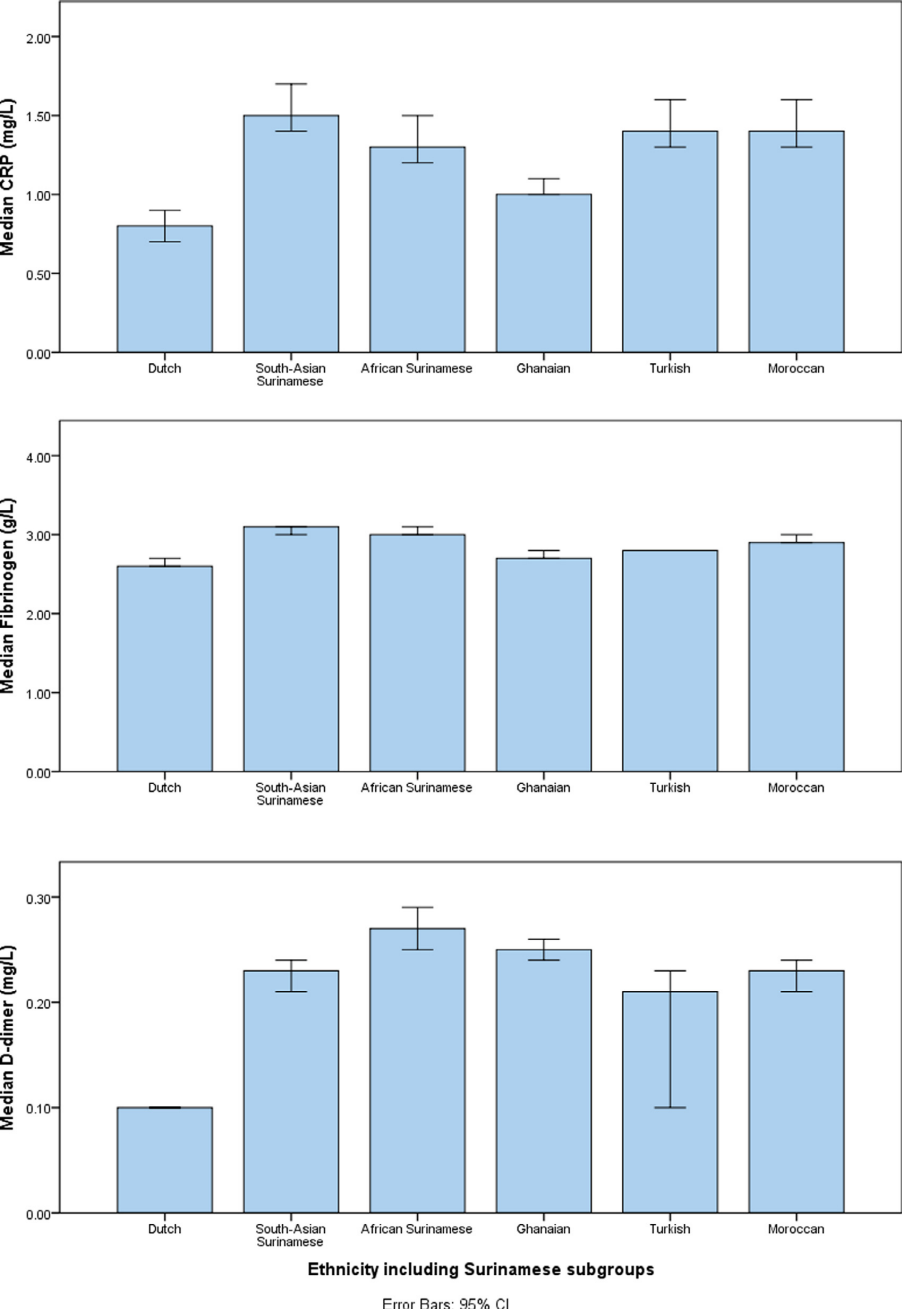
Concentrations of inflammatory biomarkers in different ethnic groups. Abbreviation: CRP = C-reactive protein.

The median concentrations of the inflammatory biomarkers differed across categories of ethnicity (Fig. 1). Dutch individuals had significantly lower median hs-CRP (*p* < 0·001 for each ethnic group), and fibrinogen (*p* < 0·001 for African Surinamese, South-Asian Surinamese, Moroccans, and Turkish; *p* = 0.019 for Ghanaians).concentrations than each of the minority ethnic groups, Dutch individuals also had significantly lower median D-dimer concentrations than the South-Asian Surinamese (*p* < 0·001), African Surinamese (*p* < 0·001), Ghanaian (*p* < 0·001) and Moroccan (*p* *=* 0.001) groups, but not Turkish (*p* = 0·131). There were notable variations in the median concentrations of inflammatory biomarkers among the ethnic minority groups. For example, Ghanaians had significantly lower median concentrations of hs-CRP concentrations (*p* < 0·001) compared with the other ethnic minority groups, and significantly lower median fibrinogen concentration (*p* < 0·001) than other ethnic minority groups, except for Turkish (*p* = 1·000). In all ethnic groups, the proportion of participants with elevated hs-CRP was greater than the proportions with elevated D-dimer or fibrinogen (Table 1)

### 3.3 Associations between inflammation and vascular dysfunction

In the unadjusted model analyses of the pooled dataset, higher Z-score hs-CRP, fibrinogen, and D-dimer were significantly associated with higher odds of CAD, PAD, and aortic stiffness, except for the association between Z-score D-dimer and CAD (Table 2). In the fully adjusted model, the associations [AOR, 95% CI, *p*-value] remained positive and statistically significant for the association between Z-score hs-CRP and CAD [1·15 (1·07–1·24), *p* < 0·001], and elevated fibrinogen and CAD [1·09 (1·01–1·19), 0·034]. Similar observations were made for the associations of elevated hs-CRP, fibrinogen, and D-dimer (based on the clinically acceptable cut-off values) with CAD, PAD, and aortic stiffness (Supplementary Table 1).

**Table 2:** Associations of Z-score hs-CRP, fibrinogen, and D-dimer with macrovascular dysfunction in the whole cohort.

	Aortic stiffness		Coronary artery disease		Peripheral artery disease	
	OR (95% CI), *p*-value		OR (95% CI), *p*-value		OR (95% CI), *p*-value	
	Model 1	Model 2	Model 1	Model 2	Model 1	Model 2
hs-CRP	1.25 (1.15–1.36), <0.001	1.05 (0.94–1.16), 0.372	1.21 (1.13–1.29), <0.001	1.15 (1.07–1.24), <0.001	1.16 (1.07–1.26), 0.001	1.04 (0.95–1.15), 0.373
Fibrinogen	1.58 (1.44–1.73), <0.001	1.04 (0.93–1.16), 0.502	1.18 (1.10–1.27), <0.001	1.09 (1.01–1.19), 0.034	1.12 (1.02–1.23), 0.016	1.01 (0.91–1.12), 0.891
D-Dimer	1.15 (1.07–1.23), <0.001	0.99 (0.92–1.08), 0.881	1.05 (0.98–1.11), 0.174	1.02 (0.95–1.09), 0.666	1.07 (1.00–1.15), 0.047	1.05 (0.97–1.13), 0.235

Abbreviations: CI = confidence interval, hs-CRP = high sensitivity C-reactive protein, OR =odds ratio.

Model 1: unadjusted; Model 2: fully adjusted i.e. adjusted for age, sex; smoking (pack-years), BMI, hypertension, HbA1c, total cholesterol, and use of statins.

### 3.4 Ethnic differences in the associations between inflammation and vascular dysfunction

In the unadjusted models, different associations were observed between individual inflammatory biomarker concentrations and vascular dysfunction across ethnic groups (Table 3). For example, in all ethnic groups, except for Ghanaians, higher Z-score inflammatory biomarkers concentrations were significantly associated with higher odds of aortic stiffness. Also, higher Z-score inflammatory biomarkers concentrations were significantly associated with higher odds of CAD in all ethnic groups except South-Asian Surinamese. Further, higher Z-score inflammatory biomarkers concentrations were associated with PAD in South-Asian Surinamese, Ghanaians, and Moroccans. In the fully adjusted models, higher Z-score hs-CRP concentration was associated with higher odds of CAD among Dutch [1·63 (1·21–2·18), *p* = 0·001], higher odds of PAD among South-Asian Surinamese [1·25 (1·03–1·53), *p* = 0·024], and lower odds of PAD in African Surinamese [0·71 (0.54–0·94),*p* = 0·016]. Higher Z-score fibrinogen concentration was associated with higher odds of CAD [1·28 (1·03–1·59),*p* = 0·028] but lower odds of PAD [0·75 (0·58–0·97),*p* = 0·031] in African Surinamese individuals. Higher Z-score D-dimer concentration was associated with higher odds of PAD in Moroccan individuals only [1·39 (1·01–1·93),*p* = 0·046]. Similar patterns with varying strengths of associations were observed when elevated hs-CRP, fibrinogen, and D-dimer based on the clinically acceptable cut-off values were used instead of the Z-score values (Supplementary Table 2).

**Table 3:** Associations between Z-score inflammatory biomarker concentration and vascular dysfunction stratified by ethnicity.

	Aortic stiffness		Coronary artery disease		Peripheral artery disease	
	OR (95% CI), *p*-value		OR (95% CI), *p*-value		OR (95% CI), *p*-value	
	Model 1	Model 2	Model 1	Model 2	Model 1	Model 2
hs-CRP						
Dutch	1.37 (1.04–1.78), 0.023	0.93 (0.64–1.37),0.725	1.56 (1.20–2.03),0.001	1.63 (1.21–2.18),0.001	1.02 (0.71–1.47),0.908	1.00 (0.66–1.52),0.997
South-Asian Surinamese	1.19 (1.00–1.42),0.053	1.20 (0.96–1.51),0.111	1.14 (0.98–1.33),0.096	1.10 (0.93–1.32),0.266	1.27 (1.06–1.51),0.008	1.25 (1.03–1.53),0.024
African Surinamese	1.10 (0.91–1.34), 0.332	0.94 (0.73–1.21),0.629	1.23 (1.04–1.46),0.014	1.17 (0.97–1.42),0.105	0.98 (0.79–1.22),0.863	0.71 (0.54–0.94),0.016
Ghanaian	1.22 (0.98–1.52), 0.072	0.95 (0.74–1.24),0.721	1.17 (0.96–1.43),0.121	1.20 (0.97–1.50),0.097	1.31 (1.06–1.61),0.012	1.12 (0.88–1.43),0.354
Turkish	1.35 (1.11–1.65), 0.003	1.12 (0.85–1.47),0.434	1.12 (0.97–1.30),0.126	1.11 (0.94–1.32),0.214	1.16 (0.96–1.40),0.126	1.10 (0.89–1.38),0.374
Moroccan	1.27 (1.05–1.54), 0.016	1.16 (0.90–1.50),0.245	1.01 (0.87–1.17),0.899	0.96 (),0.81–1.140.657	0.98 (0.78–1.24),0.876	0.97 (0.74–1.26),0.795
FIBRINOGEN						
Dutch	1.93 (1.43–2.60), <0.001	0.89 (0.62–1.27),0.529	1.29 (0.94–1.76),0.114	1.26 (0.88–1.80),0.208	0.97 (0.71–1.34),0.866	0.92 (0.64–1.34),0.677
South-Asian Surinamese	1.64 (1.35–1.99), <0.001	1.25 (1.00–1.58),0.055	1.06 (0.90–1.25),0.504	0.90 (0.74–1.08),0.249	1.05 (0.86–1.28),0.614	0.98 (0.78–1.23),0.878
African Surinamese	1.35 (1.09–1.68), 0.005	0.93 (0.72–1.21),0.592	1.33 (1.10–1.61),0.003	1.28 (1.03–1.59),0.028	0.99 (0.80–1.24),0.958	0.75 (0.58–0.97),0.031
Ghanaian	1.21 (0.97–1.51), 0.094	0.80 (0.61–1.04),0.099	1.29 (1.06–1.57),0.011	1.25 (0.99–1.56),0.056	1.11 (0.89–1.39),0.351	0.90 (0.70–1.16),0.420
Turkish	1.93 (1.50–2.49), <0.001	1.32 (0.97–1.79),0.074	1.03 (0.87–1.23),0.711	0.97 (0.80–1.17),0.727	1.23 (0.98–1.53),0.071	1.09 (0.85–1.40),0.488
Moroccan	1.49 (1.16–1.91), 0.002	0.95 (0.69–1.30),0.731	0.97 (0.82–1.15),0.749	0.89 (0.73–1.08),0.232	1.06 (0.82–1.38),0.654	1.06 (0.78–1.43),0.725
D-DIMER						
Dutch	1.18 (0.99–1.40), 0.070	0.89 (0.62–1.28),0.529	1.04 (0.80–1.36),0.764	0.95 (0.70–1.28),0.731	1.06 (0.83–1.37),0.632	1.10 (0.85–1.42),0.474
South-Asian Surinamese	1.20 (1.01–1.43), 0.040	1.00 (0.81–1.23),0.983	1.04 (0.87–1.25),0.658	0.96 (0.78–1.16),0.650	1.24 (1.04–1.48),0.015	1.21 (1.00–1.46),0.052
African Surinamese	1.10 (0.92–1.31), 0.310	0.93 (0.75–1.15),0.495	1.09 (0.93–1.29),0.285	1.05 (0.87–1.25),0.619	1.11 (0.93–1.33),0.241	1.03 (0.83–1.30),0.766
Ghanaian	1.07 (0.97–1.18), 0.159	1.02 (0.91–1.13),0.791	1.04 (0.94–1.15),0.410	1.03 (0.93–1.14),0.543	0.97 (0.81–1.16),0.748	0.93 (0.74–1.18),0.574
Turkish	1.59 (1.17–2.16), 0.003	0.94 (0.61–1.45),0.786	1.13 (0.87–1.46),0.366	1.03 (0.78–1.38),0.818	0.91 (0.61–1.34),0.617	0.73 (0.46–1.15),0.177
Moroccan	1.41 (1.07–1.87), 0.016	1.18 (0.78–1.80),0.426	1.09 (0.86–1.39),0.483	1.08 (0.84–1.38),0.572	1.39 (1.03–1.86),0.029	1.39 (1.01–1.93),0.046

Abbreviations: CI = confidence interval, hs-CRP = high sensitivity C-reactive protein, OR =odds ratio.

Model 1: unadjusted; Model 2: fully adjusted i.e. adjusted for age, sex; smoking (pack-years), BMI, hypertension, HbA1c, total cholesterol, and use of statins.

Interaction analysis showed that the associations between some markers of inflammation and aortic stiffness in Dutch, South Asian Surinamese, and Turkish differed by sex. In the unadjusted models, the odds ratios for the associations between markers of inflammation and aortic stiffness were higher in males than in females in all three ethnic groups (supplementary Table 3). Albeit, the strengths of the associations were not statistically significant in the fully adjusted model for both males and females in all three ethnic groups.

## 4 Discussion

Our study shows varying levels of circulating inflammatory biomarkers (hs-CRP, fibrinogen, and d-dimer) in different ethnic groups. The levels of the individual inflammatory biomarkers were unequally associated with prevalent aortic stiffness, CAD, and PAD in different ethnic groups. The conventional cardiometabolic risk factors did not fully explain the associations between elevated inflammatory biomarkers concentrations and vascular dysfunction in the various ethnic groups.

Using the HELIUS data, we have previously reported higher hs-CRP levels among ethnic minorities living in the Netherlands, compared to Dutch individuals [11]; an observation that is consistent with other prior studies [33]. This current analysis shows that in addition to hs-CRP, the levels of clotting activation factors inherently linked with inflammation also vary among ethnic groups. Our finding of lower levels of D-dimer in Dutch participants compared with South-Asian Surinamese and Ghanaians is consistent with previous studies that have reported lower levels of D-dimer in individuals of Western European origin compared with Asian or African origin [34,35]. Our findings lend further credence to earlier studies that have reported higher levels of fibrinogen in African and South Asian individuals, compared to individuals of Western European origin [36].

The current study shows that elevated inflammatory biomarker levels were associated with CAD in Dutch, Africans from Suriname and Ghana, and Moroccans, but not in Turkish and South-Asians from Suriname; the positive associations remained statistically significant in the fully adjusted model for Dutch and African Surinamese. The findings in Dutch individuals agree with prior studies independently linking inflammation to CAD or CAD-risk in populations of Western European ethnic descent [9,37,38]. The current findings expand the existing evidence in Western Europeans to an African descent population. In Africans from Ghana and Morocco, the weakening of the strength of association after adjusting for a wide range of cardiometabolic risk factors suggests that the association between inflammation and CAD in these groups could be partly dependent on these cardiometabolic risk factors. Our observed lack of association between inflammation and CAD in South Asians from Suriname is consistent with reports by Mehta et al. that showed no association between inflammation and coronary artery calcium, a surrogate marker of subclinical atherosclerosis in the coronary circulation [39]. The lack of association between inflammation and CAD in Turkish individuals represents novel baseline data. Based on the cross-sectional design of this study, the ethnic differences in the associations between inflammation and CAD could either reflect the differential contribution of inflammation to the development of CAD or the differential dysregulation of inflammatory pathways following adverse coronary events in different ethnic groups [40]. In the case of the former, the role of elevated inflammatory biomarker levels in CAD risk prediction may vary by ethnicity. A longitudinal study may verify or refute this claim.

Similar to CAD, our study shows that the association between elevated inflammatory biomarker levels and PAD showed ethnic variations; albeit, the variation pattern was different. In the unadjusted model, elevated inflammatory biomarker levels were associated with higher odds of PAD in South Asian Surinamese and Africans from West Africa and Morocco. This study provides novel ethnic-specific data in South-Asian Surinamese and Moroccans. Our findings in Ghanaians confirm a previous report [41]; in this previous report, the observed associations between inflammation and PAD persisted in a fully adjusted model. It is worth noting that the previous report [41] included Ghanaians living in Ghana, and did not adjust for statins in the fully adjusted model; statins are known to alter inflammatory biomarker levels and their effect on cardiovascular disease [9]. Prior studies comparing the association between inflammation and PAD in Dutch individuals are lacking; previous studies including a Rotterdam study [42], were not limited to individuals of Dutch ethnicity. It was somewhat surprising that higher hs-CRP and fibrinogen levels were associated with lower odds of PAD in African Surinamese individuals, after adjusting for the conventional cardiometabolic risk factors. It remains unclear the biological basis and significance of this finding, considering the available evidence establishing inflammation as an important process for the initiation and progression of atherothrombotic diseases like PAD [9]. The fact that this observation was made for two different inflammatory biomarkers (hs-CRP and fibrinogen) limits the likelihood of chance. This unexpected inverse association could be due to a misclassification of PAD based on the recommended diagnostic criteria (ABI ≤ 0.9). This is especially true in individuals with calcified or non-compressible distal arteries, in which case the ABI is elevated [22]. A previous study has shown that African Surinamese across a wide age range have high aortic pulse wave velocities [5]. It is also possible that an ABI of ≤ 0.9 may not be a valid cutoff point for African Surinamese individuals suggesting the need for further studies to validate the cutoff in ethnic minority groups.

The current study showed a positive association between elevated inflammatory biomarker levels and the odds of aortic stiffness in the unadjusted model, although the strength of association was not statistically significant in Ghanaian individuals. Generally, our findings resemble previous reports based on populations with European and South Asian origins [43]. Adjusting for the conventional cardiometabolic risk factors attenuated the observed associations. The lack of association between inflammation and aortic stiffness in all ethnic groups limits the role of inflammatory biomarkers as risk predictors for aortic stiffness in the ethnic groups we studied. It remains unclear why elevated inflammatory biomarker levels are more often associated with CAD and PAD than in aortic stiffness, after adjustment for the cardiometabolic risk factors. Existing data, however, shows that the prognostic potency of the risk factors for atherogenesis differs in the various arterial beds [44]. Additionally, the added effects of the conventional risk factors to vascular injury may differ between arterial vascular beds [44].

In light of the influence of early childhood health-related behavior, socioeconomic status, dietary factors, and epigenetics on the predictive role of risk factors on vascular disease, it remains unclear whether findings from this study can be generalized to populations of similar ancestry but living in different environments. Additional research in this area might include the role of environmental factors on the ethnic differences in the association between inflammation and vascular dysfunction. Replicating this study in older or younger populations compared to the current study may also be valuable.

The mechanistic basis for the ethnic differences in the associations between inflammatory biomarker levels and vascular dysfunction remains uncertain. It is plausible that the vasculature of individuals of different ethnic backgrounds may exhibit different sensitivities to exposure to a given level of inflammation [13], resulting in varying degrees of vascular injuries. Alternatively, a given degree of vascular injury could manifest as different blood levels of the inflammatory biomarker in individuals from different ethnic groups, given the ethnic differences in the inflammatory response to injury [45]. Further research in this area might include the determination of vascular response to inflammation in different arterial circulations in different ethnic groups.

Our study has strengths and limitations. This study provides new important information on inflammatory biomarker levels and their associations with vascular dysfunction in a multi-ethnic population. Our study also used a population from the same city, which minimizes confounding effects from living in different cities and countries, which may have different cultural norms and lifestyles. Additionally, we used well-standardized study protocols and multiple markers of inflammation and adjusted for a wide range of covariates in our logistic regression models. Our study is limited because of its cross-sectional design and therefore we cannot exclude the possibility of reverse causation. Additionally, coronary arteriography was not performed in the evaluation of CAD, due to feasibility. Notwithstanding, pathological Q waves in contiguous leads are pathognomonic of a prior acute coronary event, regardless of symptoms [46], and correlate well with critical coronary occlusions [47]. Also, the Rose Angina Questionnaire has a high specificity to detect CAD and is valuable for screening individuals at risk of CAD in large-scale epidemiological surveys [48]. Conventional arteriography, the gold standard for vascular imaging, and other advanced imaging modalities like CT and MR angiography were not employed in the assessment of PAD due to feasibility. Albeit, ABI is known to correlate well with angiographically verified PAD [49]. Further, we did not assess the influence of dietary factors on low-grade inflammation, and its impact on vascular dysfunction. Inflammation might be one of the pathways through which diet affects insulin resistance, and possibly cardiovascular disease [50]. Assessing the influence of dietary factors could have shed some light on the mechanisms linking inflammation and vascular dysfunction in different ethnic groups. Finally, other inflammatory markers aside from CRP, fibrinogen, and D-dimers, as well as other atherosclerotic macrovascular diseases including aortic atherosclerosis, thoracic or abdominal aortic aneurysm, and carotid atherosclerosis were not evaluated in this study. This limits the generalization of our findings to all inflammatory biomarkers and measures of macrovascular disease.

In conclusion, the current study shows that inflammatory biomarker levels are unequally associated with CAD, PAD, and aortic stiffness in different ethnic groups. The associations of inflammatory biomarkers with CAD, PAD, and aortic stiffness in African populations from Ghana, Suriname, and Morocco, as well as the South Asian Surinamese show important variations from existing reports in European, African American, and North American populations. Therefore, the roles of inflammation in vascular disease risk prediction may vary between ethnic groups. Ethnicity could thus play an important role in the incorporation of biomarkers of inflammation into standard models for the prediction of cardiovascular risk. Our data provide a basis for future studies aimed at assessing the predictive roles of inflammation on vascular disease in different ethnic groups. The traditional cut-off values for defining vascular dysfunction, based on populations with European origin, may also vary in other ethnic groups and warrants future studies for ethnic-specific cut-off for vascular dysfunction including PAD and aortic stiffness. These could potentially aid vascular disease prevention, diagnosis, and treatment efforts in different ethnic groups.

## Author contributions

All authors have contributed substantially to this article and approved the submission. C.F.H-B, L.M., A.H.M, C.A., and B.B, conceived the idea; C.F.H-B., L.M., B.B, and C.A. were responsible for data acquisition; C.F.H-B, L.M, and C.A were responsible for statistical analysis. C.F.H-B, L.M, A.H.M, B.B., A.G.B.A., D.H.R, and C.A were responsible for data analysis/interpretation. Each author contributed important intellectual content during article drafting or revision and accepts accountability for the overall work by ensuring that questions about the accuracy or integrity of any portion of the work are appropriately investigated and resolved. C.F.H-B. takes responsibility for the fact that this study has been reported honestly, accurately, and transparently, that no important aspects of the study have been omitted, and that any discrepancies from the study as planned have been explained

## Data sharing statement

The HELIUS data are owned by the Academic Medical Center (AMC) in Amsterdam, The Netherlands. Any researcher can request the data by submitting a proposal to the HELIUS Executive Board as outlined at http://www.heliusstudy.nl/en/researchers/collaboration.

## Funding

The HELIUS study is conducted by the Amsterdam University Medical Centers, location AMC and the Public Health Service of Amsterdam. Both organizations provided core support for HELIUS. The HELIUS study is also funded by research grants of the Dutch Heart Foundation (Hartstichting; Grant no. 2010T084), the Netherlands Organization for Health Research and Development (ZonMw; Grant no. 200,500,003), the European Integration Fund (EIF; Grant no. 2013EIF013) and the European Union (Seventh Framework Programme, FP-7; Grant no. 278,901). We are most grateful to the participants of the HELIUS study and the management team, research nurses, interviewers, research assistants, and other staff who have taken part in gathering the data of this study.

## Declaration of Competing Interest

The authors have nothing to declare.

## Appendix Supplementary materials

Image, application 1

Image, application 2

Image, application 3
