# Supplementary material for: Inflammation and its associations with aortic stiffness, coronary artery disease and peripheral artery disease in different ethnic groups: The HELIUS Study
Source: eClinicalMedicine. 2021 Jul 7;38:101012. doi: 10.1016/j.eclinm.2021.101012 (PMC8271115; doi:10.1016/j.eclinm.2021.101012)
Supplement: Supplementary file 1 [file mmc1.docx]

Supplementary Table 1: Associations of elevated hs-CRP, fibrinogen, and D-dimer with vascular dysfunction in the whole cohort

|  | Aortic stiffness | | Coronary artery disease | | Peripheral artery disease | |
| --- | --- | --- | --- | --- | --- | --- |
|  | OR (95% CI), p-value | | OR (95% CI), p-value | | OR (95% CI), p-value | |
|  | Model 1 | Model 2 | Model 1 | Model 2 | Model 1 | Model 2 |
| hs-CRP | 1.41 (1.140-1.75), 0.001 | 0.93 (0.73-1.19), 0.558 | 1.55 (1.31-1.83), <0.001 | 1.40 (1.16-1.68), <0.001 | 1.49 (1.21-1.84), <0.001 | 1.17 (0.93-1.47), 0.186 |
| Fibrinogen | 2.54 (2.03-3.18), <0.001 | 1.17 (0.91-1.52), 0.223 | 1.50 (1.23-1.84), <0.001 | 1.27 (1.02-1.57), 0.034 | 1.38 (1.06-1.78), 0.015 | 1.06 (0.80-1.40), 0.676 |
| D-Dimer | 1.86 (1.41-2.46), <0.001 | 1.18 (0.86-1.60), 0.303 | 1.22 (0.95-1.57), 0.121 | 1.12 (0.86-1.45), 0.392 | 1.42 (1.05-1.91), 0.022 | 1.25 (0.92-1.71), 0.159 |

Abbreviations: CI = confidence interval, hs-CRP = high sensitivity C-reactive protein, OR =odds ratio

Model 1: unadjusted; Model 2: fully adjusted i.e. adjusted for age, sex; smoking (pack-years), BMI, hypertension, HbA1c, total cholesterol, and use of statins
