# Supplementary material for: Inflammation and its associations with aortic stiffness, coronary artery disease and peripheral artery disease in different ethnic groups: The HELIUS Study
Source: eClinicalMedicine. 2021 Jul 7;38:101012. doi: 10.1016/j.eclinm.2021.101012 (PMC8271115; doi:10.1016/j.eclinm.2021.101012)
Supplement: Supplementary file 2 [file mmc2.docx]

Supplementary Table 2: Associations of elevated hs-CRP, fibrinogen, and D-dimer with vascular dysfunction stratified by ethnicity

|  | Aortic stiffness | | Coronary artery disease | | Peripheral artery disease | |
| --- | --- | --- | --- | --- | --- | --- |
|  | OR (95% CI), p-value | | OR (95% CI), p-value | | OR (95% CI), p-value | |
|  | Model 1 | Model 2 | Model 1 | Model 2 | Model 1 | Model 2 |
| *hs-CRP* |  |  |  |  |  |  |
| Dutch | 1.81 (0.91-3.62), 0.091 | 0.82 (0.34-1.96), 0.654 | 3.47 (1.82-6.60), <0.001 | 4.00 (1.99-8.03), <0.001 | 1.07 (0.44-2.59), 0.873 | 0.86 (0.32-2.31), 0.761 |
| South-Asian Surinamese | 1.11 (0.71-1.74), 0.636 | 1.00 (0.59-1.68), 0.987 | 1.35 (0.94-1.96), 0.108 | 1.26 (0.84-1.90), 0.272 | 1.57 (1.02-2.42), 0.042 | 1.42 (0.88-2.29), 0.156 |
| African Surinamese | 1.40 (0.87-2.27), 0.168 | 0.90 (0.51-1.58), 0.714 | 1.84 (1.21-2.80), 0.004 | 1.61 (1.01-2.55), 0.044 | 0.97 (0.56-1.67), 0.904 | 0.51 (0.27-0.95), 0.035 |
| Ghanaian | 1.34 (0.76-2.35), 0.311 | 0.81 (0.43-1.52), 0.513 | 1.56 (0.96-2.52), 0.072 | 1.64 (0.98-2.75), 0.059 | 2.03 (1.21-3.39), 0.007 | 1.56 (0.90-2.73), 0.116 |
| Turkish | 1.70 (1.02-2.81), 0.040 | 1.19 (1.00-1.42) ,0.053 | 1.24 (0.87-1.77), 0.230 | 1.27 (0.86-1.88), 0.230 | 1.77 (1.14-2.76), 0.012 | 1.62 (0.98-2.67), 0.061 |
| Moroccan | 1.37 (0.81-2.33), 0.243 | 1.03 (0.55-1.93), 0.932 | 0.93 (0.64-1.36), 0.716 | 0.83 (0.55-1.26), 0.375 | 1.04 (0.58-1.85), 0.892 | 1.01 (0.53-1.91), 0.977 |
|  |  |  |  |  |  |  |
| *FIBRINOGEN* |  |  |  |  |  |  |
| Dutch | 2.40 (0.98-5.92), 0.056 | 0.70 (0.22-2.20), 0.544 | 2.25 (0.85-5.95), 0.103 | 2.06 (0.72-5.84), 0.175 | 1.85 (0.64-5.38), 0.259 | 2.21 (0.71-6.81), 0.169 |
| South-Asian Surinamese | 2.68 (1.74-4.15), <0.001 | 1.68 (1.01-2.79), 0.045 | 1.48 (0.98-2.22), 0.060 | 1.12 (0.71-1.75), 0.630 | 1.10 (0.66-1.85), 0.718 | 0.85 (0.47-1.51), 0.572 |
| African Surinamese | 2.27 (1.42-3.64), 0.001 | 1.36 (0.79-2.34), 0.273 | 1.62 (1.04-2.54), 0.033 | 1.45 (0.89-2.35), 0.134 | 1.23 (0.71-2.13), 0.466 | 0.79 (0.42-1.47), 0.456 |
| Ghanaian | 1.65 (0.88-3.10), 0.122 | 0.71 (0.36-1.42), 0.337 | 1.49 (0.84-2.65), 0.170 | 1.32 (0.71-2.45), 0.381 | 1.28 (0.66-2.50), 0.470 | 0.81 (0.40-1.67), 0.575 |
| Turkish | 3.77 (2.12-6.70), <0.001 | 1.61 (0.77-3.37), 0.209 | 1.09 (0.65-1.84), 0.740 | 1.03 (0.60-1.78), 0.910 | 1.81 (0.99-3.29), 0.052 | 1.39 (0.72-2.71), 0.329 |
| Moroccan | 2.06 (1.14-3.73), 0.017 | 0.89 (0.44-1.78), 0.732 | 1.17 (0.73-1.86), 0.516 | 1.05 (0.64-1.72), 0.851 | 1.06 (0.51-2.20), 0.881 | 1.06 (0.48-2.33), 0.885 |
|  |  |  |  |  |  |  |
| *D-DIMER* |  |  |  |  |  |  |
| Dutch | 3.34 (1.49-7.50), 0.003 | 1.26 (0.46-3.49), 0.651 | 1.18 (0.35-3.93), 0.789 | 0.86 (0.25-3.02), 0.818 | 1.77 (0.61-5.14), 0.295 | 1.76 (0.58-5.36), 0.318 |
| South-Asian Surinamese | 1.50 (0.80-2.82), 0.206 | 0.99 (0.49-1.98), 0.974 | 1.27 (0.73-2.24), 0.399 | 1.06 (0.59-1.91), 0.842 | 1.61 (0.85-3.02), 0.141 | 1.50 (0.76-2.95), 0.241 |
| African Surinamese | 0.87 (0.46-1.65), 0.679 | 0.57 (0.28-1.17), 0.128 | 1.33 (0.80-2.22), 0.275 | 1.17 (0.68-2.00), 0.578 | 1.37 (0.76-2.48), 0.297 | 1.00 (0.53-1.91), 0.991 |
| Ghanaian | 1.89 (1.00-3.58), 0.049 | 1.58 (0.79-3.16), 0.199 | 1.05 (0.54-2.04), 0.882 | 1.01 (0.52-1.98), 0.976 | 1.14 (0.55-2.36), 0.727 | 1.17 (0.56-2.48), 0.675 |
| Turkish | 3.24 (1.64-6.39), 0.001 | 1.67 (0.73-3.86), 0.227 | 1.27 (0.70-2.33), 0.432 | 1.18 (0.63-2.20), 0.612 | 0.75 (0.29-1.92), 0.549 | 0.59 (0.22-1.55), 0.282 |
| Moroccan | 2.71 (1.31-5.61), 0.007 | 2.12 (0.84-5.36), 0.111 | 1.20 (0.64-2.27), 0.567 | 1.25 (0.65-2.38), 0.507 | 2.21 (1.00-4.87), 0.050 | 1.81 (0.79-4.13), 0.158 |

Abbreviations: CI = confidence interval, hs-CRP = high sensitivity C-reactive protein, OR =odds ratio

Model 1: unadjusted; Model 2: fully adjusted i.e. adjusted for age, sex; smoking (pack-years), BMI, hypertension, HbA1c, total cholesterol, and use of statins.
