# Supplementary material for: Inflammation and its associations with aortic stiffness, coronary artery disease and peripheral artery disease in different ethnic groups: The HELIUS Study
Source: eClinicalMedicine. 2021 Jul 7;38:101012. doi: 10.1016/j.eclinm.2021.101012 (PMC8271115; doi:10.1016/j.eclinm.2021.101012)
Supplement: Supplementary file 3 [file mmc3.docx]

Supplementary Table 3: Associations between Z-score inflammatory biomarker concentration and aortic stiffness stratified by sex

|  | Males | | Females | |
| --- | --- | --- | --- | --- |
|  | OR (95% CI), p-value | | OR (95% CI), p-value | |
|  | Model 1 | Model 2 | Model 1 | Model 2 |
| *Dutch* |  |  |  |  |
| Hs-CRP | 1.57 (0.95-2.59), 0.080 | 1.13 (0.52-2.44), 0.751 | 1.21 (0.87-1.67), 0.253 | 0.84 (0.54-1.33), 0.467 |
| Fibrinogen | 2.25 (1.18-4.30), 0.014 | 0.89 (0.38-2.06), 0.788 | 1.71 (1.22-2.39), 0.002 | 0.84 (0.55-1.28), 0.418 |
|  |  |  |  |  |
| *South Asian Surinamese* |  |  |  |  |
| Fibrinogen | 1.79 (1.26-2.53), 0.001 | 1.48 (1.00-2.20), 0.051 | 1.43 (1.12-1.83), 0.004 | 1.16 (0.87-1.54), 0.312 |
| D-dimer | 1.31 (1.01-1.69), 0.040 | 1.11 (0.83-1.47), 0.483 | 1.05 (0.80-1.37), 0.751 | 0.94 (0.65-1.36), 0.736 |
|  |  |  |  |  |
| *Turkish* |  |  |  |  |
| Fibrinogen | 2.92 (1.43-5.94), 0.003 | 1.85 (0.83-4.12), 0.131 | 1.63 (1.23-2.17), 0.001 | 1.15 (0.83-1.61), 0.397 |
| D-dimer | 1.58 (0.51-4.86), 0.424 | 0.74 (0.11-5.02), 0.754 | 1.32 (0.94-1.85), 0.107 | 0.95 (0.60-1.51), 0.821 |

Abbreviations: CI = confidence interval, hs-CRP = high sensitivity C-reactive protein, OR =odds ratio

Model 1: unadjusted; Model 2: fully adjusted i.e. adjusted for age, sex; smoking (pack-years), BMI, hypertension, HbA1c, total cholesterol, and use of statins
